# Adrenocortical Tumors in Children With Constitutive Chromosome 11p15 Paternal Uniparental Disomy: Implications for Diagnosis and Treatment

**DOI:** 10.3389/fendo.2021.756523

**Published:** 2021-11-05

**Authors:** Emilia Modolo Pinto, Carlos Rodriguez-Galindo, Catherine G. Lam, Robert E. Ruiz, Gerard P. Zambetti, Raul C. Ribeiro

**Affiliations:** ^1^ Department of Pathology, St. Jude Children’s Research Hospital, Memphis, TN, United States; ^2^ Department of Oncology, St. Jude Children’s Research Hospital, Memphis, TN, United States; ^3^ Department of Global Pediatric Medicine, St. Jude Children’s Research Hospital, Memphis, TN, United States

**Keywords:** Beckwith-Wiedemann syndrome, adrenocortical cancer, hemihypertrophia, chromosome 11p15, TP53, UPD

## Abstract

Pediatric adrenocortical tumors (ACTs) are rare and heterogeneous. Approximately 50% of children with ACT carry a germline *TP53* variant; however, the genetic underpinning of remaining cases has not been elucidated. In patients having germline *TP53* variants, loss of maternal chromosome 11 and duplication of the paternal copy [paternal uniparental disomy, (UPD)] occurs early in tumorigenesis and explains the overexpression of *IGF2*, the hallmark of pediatric ACT. Beckwith-Wiedemann syndrome (BWS) is also associated with overexpression of *IGF2* due to disruption of the 11p15 loci, including segmental UPD. Here, we report six children with ACT with wild type *TP53* and germline paternal 11p15 UPD. Median age of five girls and one boy was 3.2 years (range 0.5-11 years). Two patients met the criteria for BWS before diagnosis of ACT. However, ACT was the first and only manifestation of paternal 11p15 UPD in four children. Tumor weight ranged from 21.5 g to 550 g. Despite poor prognostic features at presentation, such as pulmonary metastasis, bilateral adrenal involvement, and large tumors, all patients are alive 8-21 years after cancer diagnosis. Our observations suggest that children with ACT and wild type *TP53*, irrespective of their age, should be screened for germline abnormalities in chromosome 11p15.

## Introduction

Pediatric adrenocortical tumors (ACTs) are associated with germline *TP53* mutations in 50% of cases (1, 2). For ACT cases without a germline *TP53* alteration, germline abnormalities at chromosome 11p15 loci, typically seen in the Beckwith-Wiedemann syndrome (BWS, OMIM 130650) (3–8), have been reported (1). BWS is a pediatric overgrowth and cancer predisposition syndrome. The clinical presentation is highly variable and viewed as a spectrum of *classical* (macroglossia, anterior abdominal wall defects, and prenatal and post-natal overgrowth), *atypical* (patients with isolated features of BWS) and *isolated lateralized overgrowth* (3). Affected individuals are usually born macrosomic and develop rapid growth starting either at birth or before the first year of life (3, 4). However, asymmetry may not be apparent at birth, and overall signs of overgrowth may appear subtle. Patients with BWS are also at risk of having early onset tumors such as Wilms tumor, hepatoblastoma, ACT, and neuroblastoma which are considered to originate from dysregulation of cellular processes during early embryogenesis (3–8). The variability in phenotype is due to genetic and epigenetic alterations of chromosome 11p15, with specific gene mutations, chromosomal copy number changes, or methylation status of chromosome 11p15 imprinting centers, leading to dysregulation of specific genes affecting growth, development, and cancer (5–8). Cancer risk depends on the genetic/epigenetic defect. Segmental paternal 11p15 paternal uniparental disomy (UPD) accounts for 20% of cases of BWS (3), and a 25-30% cancer risk including Wilms tumor, hepatoblastoma and ACTs (5–8). Most of the cases clinically defined as BWS who develop ACT have germline UPD (7, 8). However, since 11p15 UPD occurs as a somatic mosaic event, the true incidence of UPD might be higher than that reported in literature.

**Figure 1:**
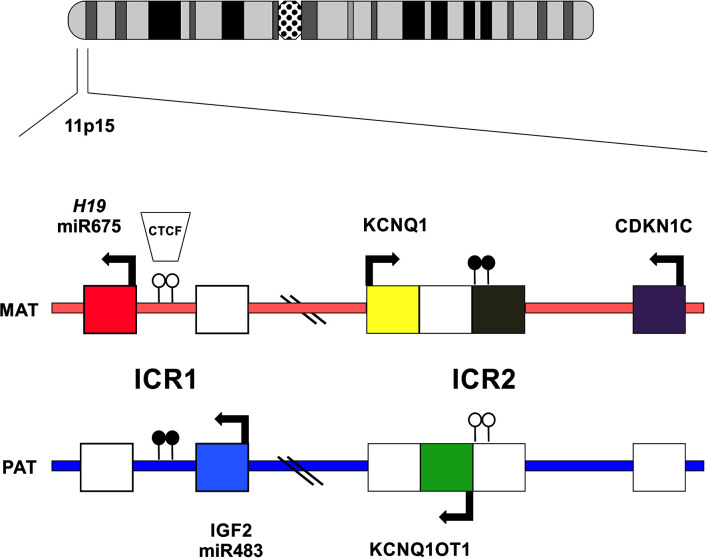
Schematic representation of imprinted gene cluster on chromosome 11p15. Genes and their directions of transcription are shown. Maternally or paternally expressed genes are indicated by filled squares. Open circles show the location on normally unmethylated ICR and filled circles indicate normally methylated ICR.

Chromosome 11p15 contains a cluster of imprinted genes important for the control of fetal and postnatal growth (**Figure 1**). The telomeric domain includes the long non-coding RNA *H19*, which is maternally expressed in the embryo and placenta (9) but silenced in most tissues after birth except in cardiac and skeletal muscles (10). Also contained within this domain is *IGF2*, which encodes, a growth factor paternally expressed in the fetus and placenta, and biallelically expressed in the liver after birth (11). The ICR1 (imprinting control region) located upstream of the *H19*, is a methylation sensitive chromatin insulator that in conjunction with enhancers, modulates the transcription of *IGF2* and *H19* in an allele-specific manner. The ICR1 is usually unmethylated in the maternal allele and therefore allows the binding of CTCF (CCCTC-binding factor), a zinc finger protein with insulating activity, thereby preventing the expression of *IGF2*, and allowing transcription of *H19* by downstream enhancers on the maternal chromosome (**Figure 1**). Conversely, the ICR1 is methylated on the paternal allele which interferes with CTCF binding, thus silencing *H19* and allowing *IGF2* expression *via* access to enhancers (12) (**Figure 1**). The centromeric ICR2 is located at the 5′ end of long non-coding RNA *KCNQ1OT1*(antisense transcript of *KCNQ1*) and mediates the silencing of several genes, including *CDKN1C*, which encodes the G1 cyclin-dependent kinase inhibitor (p57^KIP2^), that negatively regulates cell growth and proliferation. *CDKN1C* is maternally expressed in the embryo and placenta as well as postnatally throughout the body (13) (**Figure 1**). *KCNQ1* is initially maternally expressed during early embryogenesis but is then biallelically expressed during development (14). On the maternal chromosome, ICR2 is methylated, *KCNQ1OT1* is not transcribed, and the flanking imprinted genes (*KCNQ1* and *CDKN1C*) are expressed. On the paternal chromosome, the *KCNQ1OT1* promoter is not methylated, the transcript is expressed in the opposite direction of *KCNQ1*, and silences *in cis* genes of the centromeric domain on the paternal chromosome (15) (**Figure 1**).

In this study, we describe the clinical and molecular findings of six pediatric cases of ACTs with germline paternal 11p15 UPD and discuss the implications of these findings for the management of children with ACT associated with these genetic abnormalities.

## Patients and Methods

### Case Selection

Six pediatric patients with ACT, all with wild type *TP53*, were selected from the International Pediatric Adrenocortical Tumor Registry (IPACTR) at St. Jude Children’s Research Hospital (St. Jude). Written informed consent was obtained from parents or legal guardians for inclusion in the St Jude Children’s Research Hospital (St. Jude) International Pediatric Adrenocortical Tumor Registry (IPACTR; http://clinicaltrials.gov/show/NCT00700414). One patient (#1) was diagnosed with BWS at the time of cancer diagnosis. A second patient (#2) developed lateralized overgrowth and the ACT was found when surveilling for abdominal tumors. No features of BWS were seen during cancer diagnosis for the remaining four cases (#3,4,5,6) but patients were subsequently found to harbor constitutional chromosome 11p15 alterations (**Table 1**).

**Table 1:** Clinical findings of pediatric adrenocortical patients included in this study.

Case	Gender	Age (yrs)	Clinical Presentation	Additional findings	Tumor weight (g)/side	Pathologic diagnosis	Ki-67 LI	p53	*CTNNB1*	Inhibin-α	Treatment	Status (yrs)
1	F	8.5	BWS	Bilateral adrenal masses/Bilateral breast masses	21.5 (left)	ACA	<2%	<2%	WT	Subset positive	Surgery	Alive (18)
2	M	2.4	BWS	Hepatic mass	56 (left)	ACC	<5%	Negative	ND	Negative	Surgery	Alive (13)
3	F	0.5	Cushing syndrome		>100* (left)	ACC	30%	<1%	S45P	Ocassional cells	Surgery	Alive (8)
4	F	1.3	Routine visit	Bilateral pulmonary nodules	130 (left)	ACC	ND	ND	WT	ND	Surgery + Chemotherapy	Alive (28)
5	F	4	Abdominal pain	Tumor extension into the inferior cava and right atrium	550 (right)	UMP	low	20%	positive (IHC)	Negative	Surgery + Chemotherapy	Alive (21)
6	F	11	Hypertension	Tumor rupture during surgery	388 (left)	UMP	<5%	Occasional area	G34E	Negative	Surgery	Alive (23)

*Weight estimated from tumor volume (276cm^3^). BWS, Beckwith-Wiedemann syndrome; ACA, adrenocortical adenoma; ACC, adrenocortical carcinoma; UMP, uncertain malignant potential; ND, not determined.

Partial results (patients #1,3,4 and 6) has been previously published (1, 9).

### Molecular Analysis

Due to the complexity of the chromosome 11p15 imprinting regions and their interactions, the interpretation of copy number variations (16) and epigenetic changes (6, 16) in that region requires a series of molecular assays. In this series of 6 patients, whole genome or whole exome sequencing was examined for three (#1,5,6) patients and single nucleotide polymorphism array was offered to patient #3.

**Figure 2:**
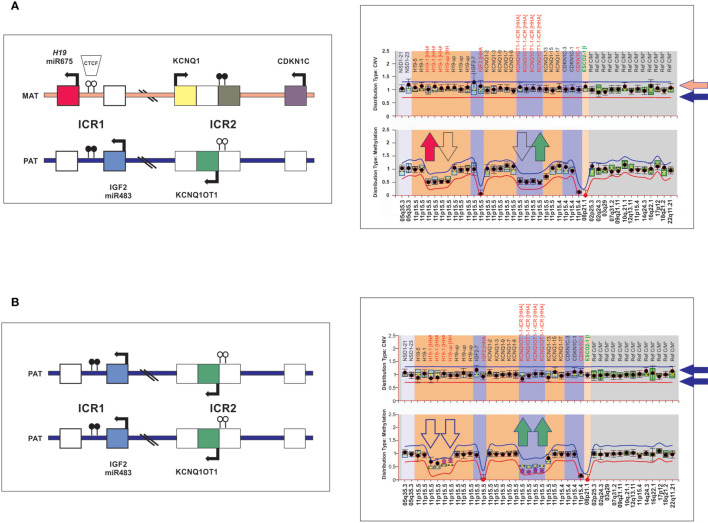
Schematic representation of the chromosome 11p15 covered by the MS-MLPA assay. **(A)** The left panel shows a diagram of the imprinted gene cluster on chromosome 11p15 with normal chromosomal copy number and methylation status. The right panel, MS-MLPA showing a diploid content of chromosome 11p15 (upper panel) and ICR1 and ICR2 for methylated probes (lower panel) in the normal range, consistent with the presence of maternal and paternal chromosomes 11p15. **(B)** Individuals with paternal 11p15 UPD as visualized by MS-MLPA. ICR1 hypermethylated and ICR2 hypomethylated consistent with loss of maternal chromosome 11p15.

Targeted techniques were combined to determine 11p15 status in germline DNA for all 6 patients. Genotyping of a panel of five microsatellite markers (D11S1363, D11S922, D11S4046, HUMTH01 and D11S988) covering positions 1,061,991 to 4,539,851 at chromosome 11p15 (GRCh37/hg19) was performed by using a fluorescently labeled forward and conventional reverse primers as previously described (1). An informative result allows us to discriminate maternal and paternal alleles for each marker. A pattern of homozygosity is suggestive of paternal uniparental disomy (UPD). In addition, genomic DNA was analyzed by using a methylation-specific multiplex ligation-dependent probe amplification (MS-MLPA; ME030-C3 BWS/RSS, MRC-Holland), commercially designed specifically for the 11p15 region and currently the most rapid and robust technique to assess methylation. DNA was processed in parallel with and without digestion with the methylation sensitive *Hha*I enzyme to detect both chromosome copy number alteration and methylation deregulation. Data analysis was performed using the Coffalyser software (MRC Holland) which provides two outputs, one related to chromosome 11p15 copy number changes and the other methylation status of imprinting control 1 (ICR1; regulating *H19* and *IGF2*; position 1,976,280 to 1,982,450) and ICR2 (regulating *CDKN1C*, *KCNQ1* and *KCNQ1OT1*; position 2,677,130 to 2,678,030) by the ratio of digested to undigested DNA. DNA from control individuals shows reduction of 50% of the MS-MLPA signal, corresponding to the presence of methylated alleles (ICR1 is unmethylated in maternal allele and ICR2 unmethylated in the paternal allele) and the contribution of both parental chromosomes (**Figure 2A**). Patients with UPD exhibited hypermethylation at ICR1 and hypomethylation at ICR2 consistent with the absence of maternal chromosome and duplication of paternal chromosome (**Figure 2B**). These epigenetic and structural changes at chromosome 11p15 leads to biallelic expression of *IGF2* and inactivation of *H19* and *CDKN1C*.

### Histological and Immunohistochemistry Studies

**Figure 3:**
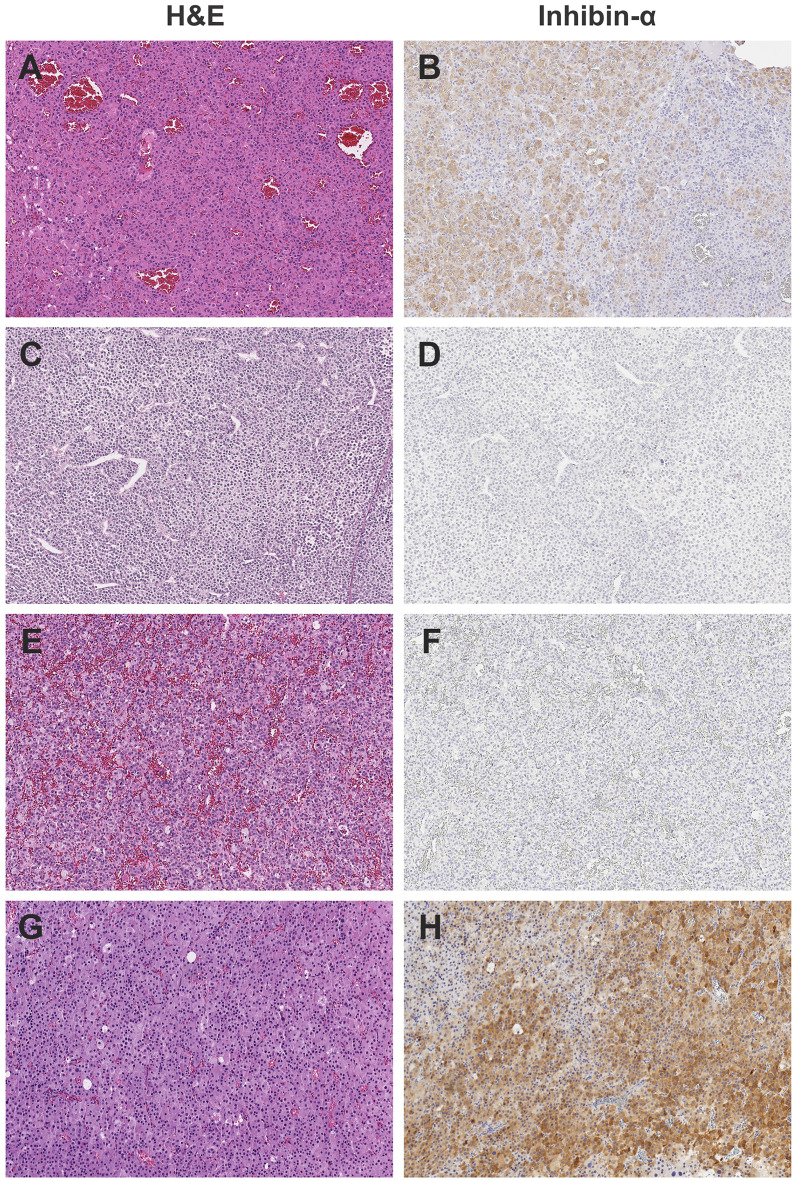
Representative histology of study cases, showing the heterogeneous appearance of adrenocortical tumors by H&E staining and weak to absent inhibin staining. All images photographed at 10x magnification using Leica Biosystems Aperio ImageScope. **(A)** H&E, **(B)** Inhibin, positive in subset of cells of Case #1; Adrenocortical adenoma. **(C)** H&E, **(D)** Inhibin, negative of case #3; Adrenocortical carcinoma. **(E)** H&E, **(F)** Inhibin, negative of Case #6; Adrenocortical tumor of uncertain malignant potential. **(G)** H&E, **(H)** Inhibin positive, patchy, adrenocortical carcinoma.

Enrollment on the IPACTR requires central review for the diagnosis of pediatric adrenocortical tumors in individuals up to 21 years of age at the time of initial diagnosis. The histological diagnosis was based on a combination of morphologic (hematoxylin and eosin) and histochemical criteria including chromogranin, cytokeratin, Cam 5.2, inhibin (**Figure 3**), Melan-A and synaptophysin. In addition, Ki-67 and beta-catenin were analyzed using standard assays (1). The p53 expression by immunohistochemistry was performed on deparaffinized tissue sections using the avidin-biotin complex method. The slides were incubated with monoclonal antibodies directed against p53 protein (1:50, DO-7; Dako Carpinteria, CA) (**Table 1**). There was no attempt to reclassify the tumors using specific histological criteria.

## Results

### Case 1

The patient was an 8.5-year-old female with presumptive diagnosis of BWS who presented to the local physician in 2003 with new onset of herculean habitus, clitoromegaly, pubic hair, acne, and apocrine odor. Abdominal computed tomography imaging revealed synchronous bilateral adrenal masses. There was no evidence of disease elsewhere. The patient underwent laparotomy with excision of the left adrenal gland and tumor enucleation of the right gland. The left tumor weighed 21.5 g and measured 5.5 × 1.5 × 0.7 cm, and right tumor measured 1.3 × 1.2 × 1.0 cm. Sections of both tumors showed similar cellular constituents. Tumors were composed of a uniform population of cells with ample acidophilic cytoplasm and moderate anisonucleosis that recapitulated the zona reticularis, consistent with the diagnosis of adrenocortical adenoma. The p53 staining was negative and Ki-67 labeling index (LI) was less than 2%. Inhibin-α was positive in a subset of cells. No chemotherapy was indicated. The patient remained asymptomatic until 2008, and then developed bilateral small breast nodules. The right breast mass measured 3.5 × 2.5 × 2.0 cm and the left inferior breast mass measured 3.0 × 2.0 × 2.0 cm and both were surgically removed. Histology of the first mass had features of tubular adenoma and that of the left mass of juvenile fibroadenoma. The patient remained clinically stable until the age of 18 years when she was taken off the protocol.

*Molecular findings*: Blood and tumor DNA of this patient was examined by whole exome sequencing (WES) (17). Results showed that the patient had a wild type sequence for *TP53* in both germline and tumor DNA as well as wild type sequence for *CTNNB1* and *ATRX* in the tumor (1, 17). Additional findings included the somatic p.E150K variant in the imprinted *MKRN3* and the p.D155fs in the cytochrome P450 Family 17 Subfamily A Member 1, *CYP17A1.* Of note, the patient harbored a germline p.E257K variant in *EGFR* (17) and the p.M595T variant in the regulator of epigenetic gene silencing *SIRT1*. Both variants were present in the tumor in the heterozygous state (17). The variant in *EGFR* was classified as likely benign in CLINVAR (variant ID 1123013) and the *SIRT1* variant was not reported. Therefore, both variants have been reported in the Genome Aggregation Database (gnomAD) originated from unrelated individual sequences as part of population genetic studies supporting lack of biological significance for both variants in the context of adrenocortical tumors. WES analysis of blood-derived DNA did not detect 11p15 UPD. However, a pattern of homozygosity at chromosome 11p15 was verified by microsatellite markers analyses and confirmed by MS-MLPA consistent with paternal uniparental disomy (UPD) in the germline sample (**Figure 2**).

### Case 2

Patient was a 2.4-year-old male with a past medical history consistent with BWS (hemihypertrophy) who had an abdominal mass in the left adrenal gland during surveillance for abdominal tumors. In addition, a hepatic mass was noted. The patient underwent laparotomy with resection of the left adrenal gland and partial hepatectomy. The adrenal tumor weighed 56 g and measured 5.9 × 5.9 × 3.6 cm. The liver lesion weighed 73 g and measured 6.5 × 4.5 × 4.0 cm. Tumors in adrenal gland and liver were histologically identical. The immune profile of both tumors was positive for pan keratin, vimentin, synaptophysin, melan-A, and was negative for inhibin-α, chromogranin, and alpha-feto protein, anti-endomysial antibody (EMA), and calretinin. Liver and adrenal masses were also negative for p53, and Ki-67 LI was <5% in both tumors. There was vascular invasion in the adrenal tumor. Peri-aortic lymph nodes were negative. Tumor margins were free of tumor. The patient was not treated with chemotherapy and remains free of disease 11 years after the diagnosis of adrenocortical carcinoma (ACC).

*Molecular findings*: Molecular studies were done for the germline sample and the patient was wild type for *TP53.* Microsatellite analysis covering the position 1,061,991 to 4,539,851 at chromosome 11p15 (GRCh37/hg19) revealed a heterozygous pattern for all studied markers but MS-MLPA revealed a pattern consistent with mosaic paternal UPD.

### Case 3

The patient was an 8.4-year-old girl diagnosed with stage II ACC at age 7 months. In 2013, she went to the local pediatric endocrinologist as she had Cushing syndrome. Her medical history revealed that she was a full-term infant with a birth weight of 6lb 12 oz (~3kg). She did not have umbilical hernia, omphalocele, macroglossia, nevus flammeus or lateralized overgrowth. An ultrasound imaging study revealed an abdominal mass in the adrenal gland region. The mass, which measured 8.2 × 7.5 × 4.5 cm was completely resected and the diagnosis of ACC confirmed. Tumor was positive for vimentin and melan-A and inhibin-α was moderate in occasional tumor cells. The Ki-67 LI was 30%. The patient was not treated with chemotherapy and has remained disease-free.

*Molecular findings*: Blood and tumor samples were available for molecular studies. No *TP53* variants were observed in the germline or tumor (1). The tumor harbored the p.S45P missense variant in *CTNNB1* and was wild type for *ATRX*. Whole genome SNP microarray (Reveal; Integrated Genetics) analysis of germline DNA revealed normal chromosome copy number but 50% mosaicism for chromosome 11p covering a 45.6 Mb region. A pattern consistent with mosaic paternal UPD was confirmed by MS-MLPA.

### Case 4

Patient was a 28-year-old healthy female diagnosed with non-functional metastatic ACT at age 15 months. She did not have an antecedent of endocrine manifestations, and the tumor was discovered during the one-year routine visit to the pediatrician. She did not have a history of growth and developmental abnormalities. CT imaging of the abdomen and chest revealed a left supra-renal mass measuring 5 cm in the largest diameter and bilateral pulmonary nodules (Stage IV). She underwent resection of the primary tumor that measured 7.0 × 6.0 × 4.0 cm and weighed 130 g. The histopathological examination confirmed the diagnosis of ACC. She was transferred to St. Jude and treated with cisplatin, etoposide and mitotane. However, after four courses of chemotherapy, several pulmonary lesions remained. She underwent resection of the bilateral pulmonary metastatic nodules, and most of them showed viable metastatic tumor by histology. Chemotherapy was stopped and the patient was monitored for tumor progression. The pulmonary lesions regressed and remained very small for over 10 years. She continues to be free of disease and in good health to date, 26.5 years after the diagnosis.

*Molecular findings*: No *TP53* variants were observed in blood and tumor samples (1). In addition, the primary tumor was negative for *CTNNB1* and *ATRX*. Furthermore, microsatellite analysis of germline DNA revealed a pattern of homozygosity consistent with paternal UPD that was confirmed by MS-MLPA.

### Case 5

The patient was a 21-year-old female with past history of stage 3 ACT diagnosed at 4.9 years. Her development was normal except for an incidental note per the family of longer and larger right upper and lower extremities throughout her life that had not been specifically investigated as far as the patient and her family were aware, but was associated with chronic left-leaning posture and asymmetric shoe size. She had been in her usual state of good health until the age 4 years when she complained of abdominal pain. She also had weight loss, increased headaches, decreased activity and energy, increased tiredness, and sweating. Given the persistence of symptoms, she was examined by a physician who palpated an abdominal mass and noted hypertension and slight androgen elevations. Magnetic resonance imaging showed a large abdominal mass in the right adrenal gland, with direct extension into the inferior vena cava and nearly completely filling of the right atrium. She underwent a partial sternotomy, and extraction of the caval portion of the adrenal tumor and resection of the right retroperitoneal tumor. The tumor weight was 550 g and measured 13.5 × 10.5 × 6.0 cm. The pathology confirmed ACC (low Ki-67 LI, rare mitotic figures and negative for inhibin-α). There were large areas of necrosis, and vascular invasion with tumor penetration through the capsule. Immunohistochemistry analysis was negative for p53 and positive for beta-catenin. She was treated with the combination of cisplatin, doxorubicin, etoposide, and mitotane. Features of right-sided lateral overgrowth, including right-sided tongue enlargement as well as increased right *vs*. left arm circumferences (51 cm *vs* 50 cm), calf circumferences (39 cm *vs* 37 cm), and a slight leg length discrepancy (88 cm *vs* 85 cm) were first noted during exams performed at St. Jude as part of a multidisciplinary rare endocrine tumor clinic when she was 18 years old, and led to additional testing subsequently confirming chromosome 11p15 UPD. She has been alive and tumor free for 17 years.

*Molecular findings:* Blood DNA of this patient was analyzed by whole exome sequencing (WES). No reportable structural, single nucleotide variants, and indel or copy number changes were identified in *APC*, *CDKN1C*, *MEN1*, *PRKAR1A*, and *TP53*. Chromosome paternal 11p15 UPD was confirmed by MS-MLPA.

### Case 6

The patient was a 23-year-old female who had hypertension during a routine medical visit in 2009 at age 11 years. Examination for causes of hypertension revealed a large, and well-defined mass in the left upper quadrant above the left kidney and deep to the spleen. The mass was approximately 8.7 × 9.7 × 9.7 cm and was relatively round. The kidneys and liver were normal in appearance. There was no regional adenopathy. Laboratory investigation showed remarkably elevated levels of deoxycorticosterone, aldosterone, and dehydroepiandrosterone. The mass was removed one month later, and weighed 388 g and measured 11 × 10 × 6 cm. There was a 3 × 3 cm defect on the surface suggestive of tumor rupture. The tumor was composed of nests of moderate-sized cells with round nuclei, prominent nucleoli, and moderate to abundant foamy to amphophilic non-foamy cytoplasm. There was only one mitosis per ten 400× fields and there was no atypical mitosis. However, the tumor demonstrated prominent venous invasion, necrosis, and extracapsular extension. Calcifications were noted. Immunohistochemical stains were positive for melan-A, vimentin, and cytokeratin (focal). Epithelial membrane antigen, chromogranin, and inhibin-α were negative. Ki-67 LI was <5%. Occasional areas of the tumor showed moderate nuclear staining for p53. The patient was referred to St. Jude for treatment. At that time, her adrenal hormones had returned to normal levels. She underwent comprehensive imaging studies, including FDG-PET scans. There was no evidence of residual disease. The recommendation was observation. She remains disease free 12 years from diagnosis.

*Molecular findings:* Blood and tumor DNA of this patient was analyzed by whole genome sequencing (WGS) (17). The patient had a wild type sequence for *TP53* in both germline and tumor DNA (1, 17). ACT was wild type for *ATRX* and acquired the p.G34E variant in *CTNNB1* and p.R201H in *GNAS* (17). In addition, the patient harbored the germline p.R307* variant in the PDE11A (18). WES analysis of blood-derived DNA was not sufficiently sensitive to detect chromosome 11p15 UPD, but MS-MLPA revealed a mosaic pattern of paternal 11p15 UPD.

## Discussion

This study suggests that ACT can be the first and only clinical manifestation of germline paternal uniparental disomy (UPD) at chromosome 11p15. The association between chromosome 11p15 rearrangements and embryonal tumors, including pediatric ACT, in patients with clinical diagnosis of BWS/lateralized overgrowth is well established (3–8). Wilms tumor is the most common tumor (52% of all tumors) (3), and pediatric ACT accounts for a minority of cases (3%) (3). The risk for embryonal tumors in BWS results primarily from dysregulation at the telomeric domain of 11p15 (gain of methylation at ICR1 and UPD) rather than at the centromeric domain (loss of methylation at ICR2 and pathogenic variants in *CDKN1C*) (19, 20). The loss of methylation at the ICR2 confers a low risk of developing embryonal tumors (19, 20). ACTs have been reported in a BWS patient with concomitant neuroblastoma and hypomethylation at ICR2 (21). However, most studies associate ACT exclusively with 11p15 UPD (19, 22, 23). In addition to paternal 11p15 UPD, germline rearrangements at this region have also been observed in 12% of children with ACT without germline *TP53* mutations (1), making abnormalities in these loci the most common recurrent constitutional driver events in wild type *TP53* pediatric ACT.

Remarkably, four of the patients with germline paternal 11p15 UPD (cases #3,4,5,6), and three previously reported cases of ACT with hypomethylation at ICR2 (24, 25) did not have clinical manifestations of BWS. These findings suggest that chromosome 11p15 abnormalities are associated with diverse phenotypes ranging from cases that fulfil the entire criteria of classic BWS to those with embryonal tumors-only. This concept is supported by a study reporting that among 437 cases of non-syndromic Wilms tumor, 13 (3%) had constitutional 11p15 abnormalities, including 6 with germline paternal segmental 11p15 UPD (26). Although mechanisms underlying the phenotypic variations are not established, the length of the uniparental disomic region has been implicated in the degree of severity (27). This is consistent with molecular findings observed in three of the patients (cases #1,5,6) included in this study. In these patients, microsatellite and MS-MLPA analysis detected mosaic paternal 11p15 UPD that was not detected by WGS or WES. These findings highlight the technical challenges of detecting genetic mosaicism and underscore the need to incorporate multiple techniques with higher diagnostic yield to determine the (epi)genotype-phenotype associated to cancer risk.

A diversity of adrenal gland lesions is observed in BWS, including adrenal hyperplasia, hemorrhage, cysts, neuroendocrine tumors, neuroblastoma, hemangioendothelioma, ovarian thecal metaplasia, and adrenal cortical tumors (adenoma and carcinoma) (28). Moreover, ectopic adrenal tissue (29) has been found in liver, renal hilum, and spinal cord. A panel of markers (CD56, vimentin, melan-A, inhibin-α, synaptophysin, chromogranin and SF-1) are used to establish the origin of adrenal tumors (30, 31). Notably inhibin-α is expressed in the normal adrenal cortex and frequently in both adrenocortical adenoma and carcinoma, which is useful to differentiate adrenal cortex tumors from other embryonal pediatric tumors. Normal adrenal glands show strong immunoreactivity against the inhibin- α subunit, especially in the zona reticularis (32, 33) and both fetal and definitive zones of the fetal cortex (32, 33). In addition, immunopositivity is seen in most ACTs (33). In our series, inhibin-α was negative in three cases and had weak focal expression (occasional cells) in two cases. Focal immunoreactivity for inhibin-α was also seen in heterotopic ACT in a patient with BWS (29). However, the clinical and pathological significance of immunoreactivity for inhibin α remains unclear.

*IGF2* expression is increased in virtually all cases of pediatric ACT, irrespective of the *TP53* status, and is considered an early driver event in tumorigenesis of the adrenal cortex (17). However, the mechanism of *IGF2* overexpression, and likely its degree, may vary. Most cases of ACT in carriers of germline *TP53* variants, have lost the whole maternal chromosome 11, followed by duplication of the paternal [copy neutral loss of heterozygosity (LOH)] (17). In this context, there is biallelic *IGF2* expression and loss of *IGF2* regulatory elements located in maternal chromosome 11. In contrast, in ACT of children with germline paternal 11p15 UPD, rearrangements appear to be confined to the 11p15 loci and *IGF2* expression could still be regulated by remaining regulators on maternal chromosome 11. Moreover, in ACT driven by *TP53* germline mutations, there is an association between tumor weight and the number of genomic alterations (17). It is not surprising that tumor size is a reliable prognostic indicator in patients with germline *TP53* variants, in which patients with small tumors (<100 g) have excellent outcomes (34–36) whereas those with large tumors can have metastatic disease at diagnosis and a high relapse rate after complete resection. These observations are consistent with the proposed mechanism of pediatric ACT associated with germline *TP53* variants that lose both the wild type *TP53* and maternal chromosome 11 early in tumorigenesis (17). As the tumor grows, these two molecular events are followed by acquisition of multiple genomic abnormalities and poor clinical outcomes in patients (17). Conversely, it appears that tumor weight in cases of ACT with constitutional paternal 11p15 UPD is not associated with high number of genomic aberrations as seen in carriers of *TP53* variants even when the former has larger tumors. In our series, all six patients with ACT associated with paternal 11p15 UPD are alive and free of disease despite bilateral pulmonary metastasis at diagnosis in one patient, bilateral tumors in another patient, and tumor extending and nearly filling the right atrium in a third patient. Of note, independent of the germline *TP53* status, copy neutral LOH with preferential loss of maternal chromosome 11 leading to *IGF2* overexpression from the paternal allele is observed in 90% of pediatric ACTs (17). Although not related to prognosis in children, chromosome 11p15 abnormalities and IGF2 overexpression are malignancy markers in adult patients with ACC (37).

Recommendations for cancer surveillance of children with BWS have been suggested by consensus panels (5, 38). For non-syndromic children with paternal UPD 11p15 who develop ACT, it is unclear whether surveillance is required beyond that for relapsed ACT. In our series, no patients developed other embryonal tumors. However, case #1, who had a clinical diagnosis of BWS preceding the diagnosis of ACT, developed bilateral breast fibroadenomas at age 14 years. This complication is seen in women with BWS (39). Until more data are available, we suggest that patients with ACT associated with paternal UPD 11p15 follow the surveillance recommendations for BWS (5, 38).

Case #1 was also interesting because in addition to paternal 11p15 UPD, it also carried a germline mutation in *EGFR* (p.E257K). A germline mutation in *EGFR* (p.D1080N) has been reported in another patient with bilateral ACT, but without overt clinical signs of BWS. Unfortunately, in the reported case (40), chromosome 11p15 copy number changes and methylation studies were not performed to determine whether the patient did or did not have germline paternal UPD 11p15. Whether germline *EGFR* mutations contributes to pediatric ACT remains to be elucidated.

In conclusion, we demonstrate that germline paternal 11p15 UPD is a relatively common event in pediatric ACT without germline *TP53* variants or somatic manifestation of BWS. Given the therapeutic implications and tumor surveillance, we recommend using chromosome 11p15 molecular assays in routine clinical work-up of patients with pediatric adrenocortical tumors, particularly those with wild type *TP53* sequence, with genetic predisposition evaluation and counseling.

## Data Availability Statement

The raw data supporting the conclusions of this article will be made available by the authors, without undue reservation.

## Ethics Statement

The studies involving human participants were reviewed and approved by St. Jude Children’s Research Hospital. Written informed consent to participate in this study was provided by the participants’ legal guardian/next of kin. Written informed consent was obtained from the minor(s)’ legal guardian/next of kin for the publication of any potentially identifiable images or data included in this article.

## Author Contributions

All authors co-wrote the manuscript and are accountable for all aspects of the work. All authors contributed to the article and approved the submitted version.

## Funding

This work was supported by the American Lebanese Syrian Associated Charities (ALSAC), Speer Charitable Trust, and Cancer Center Support Grant CA21765. The content is solely the responsibility of the authors and does not necessarily represent the official views of the National Institutes of Health.

## Conflict of Interest

The authors declare that the research was conducted in the absence of any commercial or financial relationships that could be construed as a potential conflict of interest.

## Publisher’s Note

All claims expressed in this article are solely those of the authors and do not necessarily represent those of their affiliated organizations, or those of the publisher, the editors and the reviewers. Any product that may be evaluated in this article, or claim that may be made by its manufacturer, is not guaranteed or endorsed by the publisher.
